# Social group connections support mental health following wildfire

**DOI:** 10.1007/s00127-023-02519-8

**Published:** 2023-07-10

**Authors:** Tegan Cruwys, Emily Macleod, Timothy Heffernan, Iain Walker, Samantha K. Stanley, Tim Kurz, Lisa-Marie Greenwood, Olivia Evans, Alison L. Calear

**Affiliations:** 1 School of Medicine and Psychology, The Australian National University, Canberra, Australia; 2 UNSW School of Built Environment, Sydney, Australia; 3 School of Psychological Science, University of Western Australia, Perth, Australia; 4 Centre for Mental Health Research, The Australian National University, Canberra, Australia; 5 Melbourne Centre for Behaviour Change, University of Melbourne, Parkville, Australia

**Keywords:** Community resilience, Social identity, Natural disaster, Well-being, Multiple group memberships, Post-traumatic growth, Bushfire

## Abstract

**Purpose:**

As environmental disasters become more common and severe due to climate change, there is a growing need for strategies to bolster recovery that are proactive, cost-effective, and which mobilise community resources.

**Aims:**

We propose that building social group connections is a particularly promising strategy for supporting mental health in communities affected by environmental disasters.

**Methods:**

We tested the social identity model of identity change in a disaster context among 627 people substantially affected by the 2019–2020 Australian fires.

**Results:**

We found high levels of post-traumatic stress, strongly related to severity of disaster exposure, but also evidence of psychological resilience. Distress and resilience were weakly positively correlated. Having stronger social group connections pre-disaster was associated with less distress and more resilience 12–18 months after the disaster, via three pathways: greater social identification with the disaster-affected community, greater continuity of social group ties, and greater formation of new social group ties. New group ties were a mixed blessing, positively predicting both resilience and distress.

**Conclusions:**

We conclude that investment in social resources is key to supporting mental health outcomes, not just reactively in the aftermath of disasters, but also proactively in communities most at risk.

In the summer of 2019–2020, Australia experienced the largest wildfire disaster ever recorded in terms of its size, duration, and intensity. More than 24 million hectares were destroyed [1], equivalent to the total landmass of the United Kingdom. The fire season resulted in at least 33 fatalities, the destruction of over 3000 houses, and the death or displacement of three billion animals [2, 3]. The long-lasting mental health consequences of environmental disasters are well documented, including elevated rates of depression, anxiety, and post-traumatic stress disorder (PTSD, [4]). However, the more common trajectory following disaster is one of resilience, recovery, or even personal growth [5, 6]. We examined the capacity for social group connections to reduce distress and enable resilience 12–18 months following the 2019–2020 Australian fires.

## Wildfires and mental health

While the full extent of the mental health impact of the 2019–2020 fires is still being uncovered (but see [7, 8]), previous disaster research is instructive. A meta-analysis found that PTSD and depression are markedly higher in cohorts affected by environmental disasters including earthquakes, tsunami, hurricanes, volcanic eruptions, and fires [4]. Direct experience of wildfires is associated with an increased risk of depression, anxiety, PTSD, and substance abuse, and has additional indirect effects on mental health due to loss of income, loss of home, displacement, and loss of possessions [9–11]. Effects are most severe among those who feared for their lives or lost someone close to them to the fires [12]. For around one third of adults, significant psychological distress can persist for a decade or more [10].

Importantly though, most disaster survivors show low distress and wellbeing improves over time [10, 13]. However, mental health trajectories following a disaster cannot be adequately characterised in terms of the presence versus absence of disorder and distress. Instead, recovery is frequently marked by *positive* changes—a sense of increased resilience, personal growth, or renewed appreciation for life (e.g., [14]). More generally, mental health is best represented as two correlated but distinct components: the absence of mental illness *and* the presence of wellbeing [15–17]. This wellbeing dimension includes experiences such as a sense of flourishing, life satisfaction, and hopefulness [18]. For example, a qualitative study of 20 survivors of the 2009 fires in Victoria, Australia found that people described new social connections, new skills, and new forms of creative expression that developed due to their traumatic experience [19]. In line with this, we explored both positive and negative dimensions of mental health as part of fire recovery.

## Social groups protect mental health in times of disruption and change

Social and community factors are critical for ensuring positive mental health trajectories in the wake of traumatic events. McGuire and colleagues [20] found that social support buffered the effect of severe trauma exposure on depression and PTSD symptoms among Hurricane Katrina survivors. Among those who lost property in the 2009 Victorian fires, Gibbs et al. [21] found that poor mental health outcomes were partly attributable to the disruption to community connection caused by relocation. Survivors of these fires also subjectively identified family, social, and community supports as important to their recovery [22]. Contrasting with this finding, Gallagher et al. [23] found that only *moderate* levels of involvement in formal social groups supported mental health in this population, with no or high involvement predicting poorer outcomes. Overall though, a meta-analysis with over 88,000 people from 176 studies demonstrated that social support is one of the strongest protective factors for preventing PTSD in trauma-exposed populations [24].

However, classic models of social resources in disaster contexts have faced several criticisms. First, they typically treat social relationships and the social support that arises from them as relatively *static*. For example, the stress buffering hypothesis, while important for bringing attention to the protective role of social support, does not consider how stressful life events can *change* a person’s social relationships. This conflicts with evidence from the disaster context, where an initial mobilisation of social support can be followed by deterioration in social support and sense of community over time [25]. Indeed, changes to one’s social relationships are often key to what makes a life event stressful (see [26]). Second, classic models have tended to focus on interpersonal ties, rather than social groups. This contrasts with empirical evidence suggesting that, while both interpersonal and group-based ties are beneficial, the latter is a stronger predictor of wellbeing across diverse contexts [27, 28, 29].

The social identity model of identity change (SIMIC) addresses both weaknesses [30]. SIMIC was developed to understand the links between social resources, wellbeing, and life changes. This model builds on robust evidence from experimental studies and clinical trials for a causal link between social group memberships and better health [31, 32]. This is attributed to *social identification*—the sense of self-definition arising from membership in social groups. Because social identities are the psychological essence of group life, they are also the foundation from which other social resources flow, including social support, belonging, and a sense of agency [27, 33, 34]. SIMIC states that life changes compromise wellbeing precisely because they disrupt social identities. Further, SIMIC posits that wellbeing will be protected when people have strong social group connections– either through the *continuity* of social groups that were important to them prior to the life change, or through developing *new* group memberships.

SIMIC has received empirical support in the context of diverse challenging life transitions, including among mothers [35], retirees [34, 36, 37] and students [38]. Most of these studies focus on transitions that are positive or voluntary. A smaller body of work has examined social identity processes in the context of traumatic life events, including among refugees [39, 40], people with traumatic brain injury [41, 42], and survivors of violence and abuse [43]. This supports the link between social identities and positive post-trauma trajectories (see [44, 45] for reviews), but studies have been small in nature and often qualitative. Only one study has considered some of the SIMIC pathways in a disaster context [46], but it did not assess the role of pre-disaster social group connection.

## The current study

Our study provided the first full test of SIMIC in a disaster context, in a large sample all exposed to a Criterion A traumatic event (the first criteria for a PTSD diagnosis; [47]). Our analysis orients attention toward the role of collective social resources prior to disaster. Pre-disaster group memberships are theorised to provide the foundation for mental health recovery. Specifically, we hypothesised:

H1. Pre-disaster social group connection will predict post-disaster social group connection, specifically (a) social identification with one’s disaster-affected community, (b) continuity of existing social identities, and (c) development of new social identities.

H2. Social group connection post-disaster, including: (a) social identification with one’s (disaster-affected) community, (b) continuity of existing social identities, and (c) development of new social identities, will predict mental health.

To assess the robustness of the findings, sensitivity analyses were planned to (a) control for demographic characteristics and severity of disaster exposure, and (b) replicate the analyses among people with no mental health diagnosis pre-disaster.

## Methods

### Recruitment and design

Data were drawn from a large national survey, conducted ~ 12 to 18 months after the 2019/2020 Australian fires.^1^ Inclusion criteria were ≥ 18 years old, lived in Australia since August 2019, and able to complete the survey in English. We used four recruitment methods: advertising via social and traditional media, postal invitation to affected postcodes, paid survey panels, and student undergraduate course credit. We recruited both fire-affected people (relevant to the current paper), but also people who were smoke-affected or not affected by the fires. Overall, there were ~ 3100 respondents with usable data in the overall survey, however, our final sample were only those directly fire affected and who completed our primary measures (*N* = 627; demographics summarised in Table 1). Several measures of interest (e.g., EXITS and PTSD-8) were only administered to the fire affected subsample.

**Table 1:** Demographic characteristics (*N* = 627)

Variable	Category	Percentage (%)	*N*
Gender	Male	47.0	295
	Female	52.5	329
	Gender-diverse	0.5	3
Age	18–24	15.3	96
	25–34	13.9	87
	35–44	29.2	183
	45–54	15.2	95
	55–64	15.0	94
	65–74	9.6	60
	75 +	2.0	12
Pre-disaster annual household income (AUD)	≤ $25,999	17.1	107
	$26,000-$41,599	9.6	60
	$41,600-$64,999	15.2	95
	$65,000-$90,999	25.5	160
	$91,000-$155,999	24.2	152
	$156,000 +	8.5	53
Education	High school or less	28.4	178
	Trade, certificate, or diploma	22.3	140
	Some university	12.0	75
	Tertiary degree	37.3	234
Neighbourhood SES*	1–3 (low)	32.6	190
	4–7 (medium)	37.3	217
	8–10 (high)	30.1	175
Regionality*	Major cities	37.3	217
	Regional	58.7	342
	Remote and very remote	4.0	23
Severity of fire exposure	Low	18.2	114
	Medium	38.6	242
	High	43.2	271

*Postcode at the start of the fire season was used to derive measures of regionality and neighbourhood socioeconomic status (SES) using national data from the Socio-economic Index for Areas (SEIFA, [48] and the Index of Relative Socio-Economic Advantage and Disadvantage (IRSAD, [49]. For a minority of the sample, this postcode may not correspond to the region in which they were fire affected (e.g., due to travelling at the time of the fires, being involved in fighting fires, etc.)

### Measures

#### Severity of fire exposure

Participants answered yes or no to 18 questions covering distinct types of direct threat, disruption, or loss due to fire, involvement in fire response activities or services, and indirect exposure to fire (informed by and adapted from [12, 50, 51]). Respondents who did not endorse any of these items or whose impact of the fires was only indirect (e.g., financial) were outside our population of interest and excluded from further analyses. Items were (1) summed to create a continuous indicator (0–18) of the cumulative impact of fire that people were exposed to (used as a covariate), and (2) used to categorise participants in terms of severity of exposure: *high* if they experienced major injury, deaths of one or more loved ones, felt their life was in danger, lost their home, or had remained displaced since the fire; *medium* if they experienced evacuation, lost personal property (e.g., vehicles, shed), lost pets or farm animals, were forced to relocate, lost income, or if a loved one experienced a major injury; and *low* if they were in an area with high fire alert levels, lost one or more community buildings (e.g., child’s school, friend’s home), or were involved in fighting fires or providing a service in response to the fires.

#### Mental health measures

##### Depression

The nine-item Patient Health Questionnaire (PHQ-9 [52]; measured depression symptoms over the previous two weeks, corresponding to the DSM-5 diagnostic criteria for Major Depressive Disorder (MDD), e.g., “Tired or having little energy” rated from 0 (Not at all) to 3 (Nearly every day) (sum score 0–27; Cronbach’s α = 0.93).

##### Anxiety

The seven-item General Anxiety Disorder scale (GAD-7) measured anxiety symptoms using the same response format and timeframe as the PHQ-9 with items corresponding to the DSM-5 criteria for Generalized Anxiety Disorder [53], e.g., “Not being able to stop or control worrying” (sum score 0–21; α = 0.93).

##### Post-traumatic stress disorder (PTSD)

The eight-item Post-traumatic Stress Disorder Index (PTSD-8; [54] measured the degree to which participants experienced symptoms of intrusion, avoidance, and hypervigilance (DSM-5 criteria for PTSD), e.g., “Recurrent thoughts or memories of the event” from 1 (Not at all) to 4 (Most of the time) (sum score 4–32; α = 0.92). For purposes of describing the sample, participants were also categorised based on empirically-derived recommendations as meeting the criteria for PTSD if they endorsed at least one item in each category of intrusion, avoidance and hypervigilance symptoms, sometimes or most of the time [54].

##### Wellbeing

The World Health Organization-Five Wellbeing Index (WHO-5) [55] assessed wellbeing in the past two weeks, e.g., “I woke up feeling refreshed” from 0 (None of the time) to 5 (All of the time). In accordance with recommendations, responses were summed and multiplied by 4 to give a total score from 0 to 100 (α = 0.88).

##### Resilient coping

The four-item Brief Resilient Coping Scale (BRCS-4) [56] assessed participants’ tendency to use adaptive coping strategies to manage stress, e.g., “I look for creative ways to alter difficult situations” measured from 1 (Does not describe me at all) to 5 (Describes me very well) (sum score 4–20; α = 0.73).

##### Post-traumatic growth (PTG)

The 10-item Post-Traumatic Growth Inventory–Short Form (PTGI) [57] assessed positive personal changes after the traumatic event, e.g., “I changed my priorities about what is important in life” rated from 0 (I did not experience this) to 5 (A very great degree) (sum score 0–50; α = 0.93).

##### Mental health diagnostic history

Participants were provided with a checklist of mental disorders (depression, schizophrenia, PTSD, anxiety, obsessive compulsive disorder, alcohol use disorder, substance use disorder), as well as an open-ended option to describe any other mental health diagnoses they had received. Scoring was 1 if any diagnosis prior to the 2019–2020 fire season was endorsed, or 0 for none.

#### Social group connection

##### Multiple social identities prior to the fires

The three-item before-transition subscale of the Exeter Identity Transition Scales (EXITS; [58]), 59 measured the degree to which participants had multiple social identities pre-disaster, e.g., “I belonged to lots of different groups before August 2019” rated from 1 (Do not agree at all) to 7 (Agree completely) and averaged (α = 0.93). This scale has been validated for retrospective recall of group memberships prior to a life change (e.g., [38]).

##### Social identity continuity

The three-item continuity subscale of the EXITS [58] measured the degree to which participants were able to maintain their pre-existing social identities following the disaster, e.g., “I still belong to the same groups I was a member of before August 2019” rated from 1 (Do not agree at all) to 7 (Agree completely) and averaged (α = 0.91).

##### New social identities

The three-item gain subscale of the EXITS [58] measured the degree to which participants had established new social identities following the disaster, e.g., “I have joined one or more new groups since August 2019” rated from 1 (Do not agree at all) to 7 (Agree completely) and averaged (α = 0.95).

##### Social identification with one’s local (fire-affected) community

The validated Four Item Social Identification Scale (FISI-4; [60], [61]) assessed participants’ (post-disaster) strength of identification with their local community, e.g., “My local community is an important part of how I see myself” rated from 1 (Strongly disagree) to 7 (Strongly agree) and averaged (α = 0.90).

### Analysis plan

After describing the sample, we conducted exploratory factor analysis (EFA) with the six mental health measures. This reduced the number of outcome variables for analyses, informed by prior research suggesting that both positive and negative indicators of mental health are distinct and important. Structural equation modelling (SEM) was used to evaluate the hypotheses, enabling us to include multiple dependent variables simultaneously and estimate indirect (mediation) effects of pre-disaster social group connectedness on mental health (Model 1). Model fit was assessed using five indices from the three primary categories of fit: absolute fit indices (χ^2^, AIC, SRMR), a relative fit index (NFI) and a non-centrality-based index (CFI) (as recommended by [62]). Sensitivity analyses added covariates (Model 2) and compared the fit of model among those with versus without a pre-disaster mental health diagnosis (Models 3 and 4).

## Results

Participants were 295 men, 329 women, and 3 gender diverse people. Participants were aged from 18 to 86 years (*M* = 42.98; *SD* = 15.49). Unsurprisingly, regional areas of Australia were overrepresented among respondents (54.6%), as were remote or very remote areas (4%). Participants were distributed across all deciles of neighbourhood SES, closely mirroring national census data [48], *M* = 5.33, *SD* = 2.85. Many participants (43.4%) had a mental health diagnosis prior to the fires, most commonly anxiety (20.3%) and/or depression (24.2%), closely matching the prevalence of mental disorder in the population (estimated at 45%, [63]).

The sample were substantially affected by fire, with the majority having lost their homes, had a loved one injured or killed, or feared for their lives. Among the low exposure group, 9.7% met criteria for PTSD. Among the medium exposure group, 29.5% met the PTSD criteria. Among the high exposure group, 58.7% met PTSD criteria. These percentages are high compared to other studies on disaster survivors, including Australian fire survivors [12] and studies using the same PTSD measure [54]. This suggests that (1) this particular disaster and its aftermath, which included COVID lockdowns, were experienced as particularly traumatic, and/or (2) this study was able to reach and recruit a particularly highly affected subpopulation.

### Structure of mental health constructs

Principal components analysis was utilised with oblimin rotation, which allows for correlated factors. Barlett’s test suggested that these data were suitable for factor analysis, χ^2^(15) = 2225.54, *p* < 0.001. Communalities were adequate (initial ≥ 0.46; extracted ≥ 0.47). A two-factor solution explained 78.01% of the variance. The rotation matrix indicated that factor one (which we labelled *distress*) captured the symptoms of mental disorders (factor loadings: depression 0.93, anxiety 0.90, and PTSD 0.62) and factor two (*resilience*) captured positive indicators of mental health (wellbeing 0.89, resilient coping 0.77, and PTG 0.64;). No cross-loadings were present (> 0.38). The two factors were weakly *positively* correlated (*r* = 0.10, *p* = 0.010), which speaks to the appropriateness of simultaneously modelling both. Factor scores were used as dependent variables in the analyses that follow.

### Hypothesis testing

**Fig. 1:**
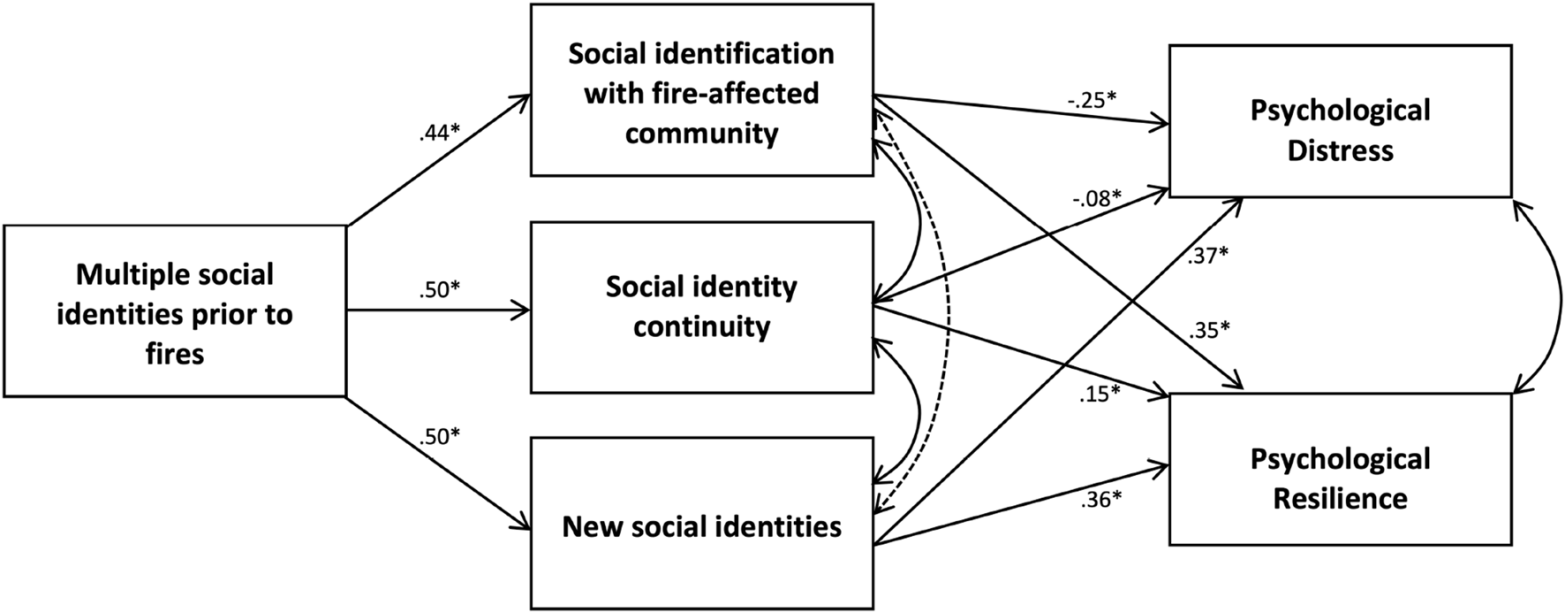
Social group connections pre- and post-disaster support mental health. Standardised beta coefficients are provided for each pathway in the model (Model 1, see text). *p < .05. Not pictured: all endogenous variables had error terms included, and covariances were specified between error terms rather than variables directly. Direct effects of pre-disaster multiple social identities on distress and resilience were not specified in the model, both because these pathways were not theorised and because this would have resulted in a fully saturated model and thus no fit indices would have been available

Table 2 reports model fit indices. Model 1 explained 15% of the variance in distress and 39% of the variance in resilience, and all pathways were significant (see Fig. 1). Consistent with H1, pre-disaster social group connectedness strongly predicted community identification (H1a), continuity of group memberships (H1b), and new social group memberships (H1c). Each of these three social connectedness indicators, in turn, significantly predicted both dimensions of the mental health. Consistent with H2a, community identification predicted less distress and greater resilience. Consistent with H2b, continuity also predicted less distress and greater resilience. However, new social identities were positively associated with both distress and resilience (providing only partial support for H2c). The indirect effect of pre-disaster multiple social identities was significant for resilience (β = 0.40, *p* = 0.019), but not distress (β = 0.03, *p* = 0.227).

**Table 2:** Fit indices

	χ^2^/*df*	SRMR	CFI	NFI	AIC
Model 1: Base model	49.71/2 = 24.86	0.039	0.96	0.95	87.71
Model 2: with covariates	30.54/2 = 15.27	0.015	0.98	0.98	158.54
Model 3: Unconstrained nested model (mental health history vs. not)	28.46/4 = 7.12	0.015	0.99	0.98	284.46
Model 4: Constrained nested model (mental health history vs. not)	49.97/13 = 3.84	0.019	0.98	0.97	287.97

*SRMR* Standardized Root Mean Square Residual (good fit < 0.08), *CFI* Comparative Fit Index (good fit ≥ 0.95), *NFI* Normed Fit Index (good fit ≥ 0.95), *AIC* Akaike information criterion (relative index: lower values indicate better fit in model comparison)

^χ2^/*df* (relative index: lower values indicate better fit in model comparison)

### Sensitivity analyses

Model 2 added covariates to the model: age, gender, income, education, and cumulative exposure. All of the covariates were allowed to covary with one another and the independent variable, and to predict all endogenous variables. Four of the covariates significantly predicted distress, and three predicted resilience: those with poor mental health were typically younger, female, lower income, and had more disaster exposure. The hypothesised pathways were qualitatively the same as the analysis without the covariates. Specifically, pre-disaster social group memberships positively predicted community identification (β = 0.40, *p* < 0.001), continuity of group memberships (β = 0.43, *p* < 0.001), and new group membership (β = 0.39, *p* < 0.001). Each of these, in turn, predicted distress (β_identification_ =  − 0.16, *p* < 0.001; β_continuity_ = -0.08, *p* = 0.037; β_new_ = 0.14, *p* < 0.001) and resilience (β_identification_ = 0.32, *p* < 0.001; β_continuity_ = 0.11, *p* = 0.001; β_new_ = 0.27, *p* < 0.001). The indirect effects of pre-disaster multiple social identities were both significant and in the hypothesised direction (β_distress_ =  − 0.05, *p* = 0.013; β_resilience_ = 0.28, *p* = 0.009).

We then added a nested structure to the covariate model, in which participants with (*N* = 272) versus without (*N* = 355) a mental health diagnostic history were separated into two groups. The unconstrained model (Model 3) fit the data in each subgroup separately. The constrained model (Model 4) required the hypothesised pathways (i.e., not covariates) to be equal in magnitude across the two subgroups. The constrained model had significantly poorer fit than the unconstrained model, Δχ^2^(9) = 21.52, *p* = 0.011, meaning that at least one pathway differed significantly in magnitude between the groups. In examining the coefficients, one pairwise parameter comparison was significant: pre-disaster multiple social identities more strongly predicted new social identities among those with a history of mental illness (β = 0.56, *p* < 0.001) than among those without (β = 0.28, *p* < 0.001). However, the relationship was significant in the hypothesised direction in both subgroups.

## Discussion

This study sought to assess the pathways through which social group connections can bolster mental health recovery in the aftermath of a disaster. Among a large sample of people substantially affected by the 2019–2020 Australian fires, validated measures evaluated mental health and collective social resources 12–18 months after the disaster. Informed by the social identity model of identity change, we hypothesised that pre-disaster social group connection would predict the quality of social group connection post-disaster, including (a) community identification, (b) continuity of existing social identities, (c) development of new social identities. We further hypothesised that each of these three indicators of social group connection post-disaster would be associated with better mental health. Hypotheses were all supported with one exception –forming new social identities post-disaster predicted greater resilience, but also greater distress. Effect sizes were substantial, and the findings were robust to different treatments of the data.

A sensitivity analysis compared those with and without a history of mental illness and found that the SIMIC model fit the data extremely well in both subgroups. This evidence increases our confidence that the findings are unlikely to be driven by effects in the reverse causal order (i.e., whereby more vulnerable or unwell respondents experienced greater decline in their social networks following the disaster). Instead, the evidence suggests that, regardless of mental health history, those who were able to maintain or even build their social group resources in the aftermath of the fires had better mental health outcomes.

However, the *positive* relationship between new social identities and psychological distress was unexpected and warrants further investigation. Post-hoc analyses with each of the six mental health measures separately bore out this finding, with new group memberships significantly predicting all six outcomes positively (both those where higher scores corresponded to greater resilience and those where higher scores corresponded to greater distress). Although most prior research has found that new group memberships are protective for health, ours is not the first to find unexpected ‘social curse’ effects (e.g., [64]. Experimental evidence has found that shared identity increases the degree to which the suffering of others is experienced as personally distressing [65]. In the context of immigration detention, shared identities were found to be mixed blessings, offering not only mutual support, but also creating a burden of others’ suffering that contributed to distress [66]. Anecdotally, many fire-affected people described forming new connections based on shared traumatic experiences, which may have had similarly mixed effects for mental health. Further analyses in our data revealed that people who had gained more new group memberships were more likely to have been severely affected by the fires, including being forced to relocate, which offers a partial explanation of these counterintuitive effects. If people are displaced by disaster, this disrupts their social group connections [67]. Some people who formed new social identities may thus have done so out of necessity. These new ties may not have been as high quality as those ties maintained from pre-disaster or those ties to local community (both of which were more straightforwardly beneficial for mental health).

### Implications

Mental health detriments of disasters are long-lasting and, in this population, continued to be pronounced 12–18 months after fire exposure. This accords with previous evidence from survivors of fire disasters in Australia [10]. Most of our sample had experienced the injury or death of a loved one, suffered substantial property damage, and/or feared for their lives, and this context is important for interpreting the extremely high prevalence of PTSD: 39% in the sample overall, but 59% in the group with the highest severity of exposure. Despite these widespread negative impacts, it is important that researchers and policymakers not lose sight of people’s remarkable capacity for resilience and even thriving following trauma. As one of the first evaluations of SIMIC in a disaster context, this study adds to a growing body of work suggesting that social identity processes are key to understanding divergent responses to trauma, including posttraumatic growth (e.g., [46, 30, 43, 68).

The possibility of such positive trajectories underscores the need for appropriate and targeted investment in social groups and communities to assist people to “build back better” following disasters (UNISDR [[69]]). Our findings elucidate the kinds of investment that may be beneficial for recovery. Recent disasters in Australia have led government to provide financial support to individuals and households. However, there has been less (and later) emphasis on investment to support social resources and communities. This study illustrates that such investment needs to be directed pre-emptively, as it is these pre-existing social resources that come to the fore in determining recovery trajectories. Such investment might consider interventions to increase social identification, which have a growing evidence base (e.g., [36, 37, 70], see [71] for a review). Moreover, it is not a matter of either investing in material support for communities or in social forms of support, as these two approaches are highly compatible [72]. While social identities are subjective, they are not independent of the real-world features of the communities in which people are embedded. For this reason, it is critical that material investments in communities, such as financial support for rebuilding infrastructure, occur in ways that are proactive and comprehensive. This contrasts with prevailing policy, which often shifts responsibility for disaster mitigation onto individuals [73], with government response to disasters being reactive and piecemeal [74]. Material investments are also likely to bolster social connection to community [75]. Furthermore, community identification provides a foundation from which to collectively and effectively organise and campaign [76, 77], including advocating for material investment in disaster preparedness, response, and recovery.

Nevertheless, it is important to be cognisant that social identities are not “one size fits all”. Not all groups are equally beneficial for mental health, and forced disruption to one’s networks (even where this presents new opportunities) may be particularly harmful. This is likely to become increasingly relevant to disaster planning and response, with estimates of up to 200 million people likely to be displaced by climate change by 2050 [78]. The evidence supports planned migration, where people affected can resettle gradually and voluntarily [79, 80]. This contrasts with forced mass migration after climate events, which affected many among the present sample. Empowering people affected by (and likely to be affected by) disasters to develop collective ownership over preparedness and recovery plans, even when they ultimately involve relocation, may protect mental health.

### Strengths and limitations

Strengths of our work include the use of validated measures and the unique and large sample of people substantially affected by fire. This allows us to be confident that the value of social group resources in protecting mental health is not limited to relatively advantaged or otherwise well populations, but is also of great relevance in communities hit by severe and lasting environmental disasters. A limitation is that our methodology cannot speak to the specific *content* of participants’ group memberships (e.g., family vs. recreational vs. professional) and thus, further research is necessary to establish whether certain group memberships are particularly helpful in a disaster recovery setting. Furthermore, we note that causal claims are not possible in the absence of experimental evidence. While caution is warranted in this regard, the measure we used to assess retrospective recall of pre-disaster group memberships has been previously found to be valid and reliable [26], and our sensitivity analyses showed that the reverse causal direction of effects was implausible. Causality could only be confidently established in future studies by trialling interventions, ideally in communities at risk of disasters, rather than in their aftermath. There is growing evidence that social identity-building interventions benefit mental health, both in intensive small-group settings and in whole populations [31, 81], [82].

## Conclusion

Climate change promises that the impact of environmental disasters will escalate in scale and scope in the coming years. Against this backdrop, this study demonstrates the crucial role of investing in cohesive communities to support disaster recovery. The more social group memberships that people affected by fires had prior to the disaster, the more they were able to maintain these groups, build new ones, and feel identified with their local (fire-affected) community. Each of these forms of social group connection were uniquely protective for mental health –both the absence of distress and the presence of resilience, with one caveat: forming new group memberships was a mixed blessing, predicting both greater resilience but also greater distress. This was likely because these new group memberships were often formed due to necessity among and between those forced to relocate by the disaster and may not have aligned with peoples’ needs. In sum, people can be remarkably resilient to the traumatic effects of disasters. However, this is only possible where those communities collectively support one another and advocate for their needs, creating a platform from which it is possible to rebuild and recover. This study demonstrates that the strength of social group ties that are present in a community *pre-disaster* produce a cascade after disaster strikes that has real impacts on mental health recovery. This speaks to the importance of government investment in *social infrastructure* as part of disaster planning: community resources that enable social group memberships.

## Data Availability

Full details of survey measures and data management for the broader research project are available here https://psychology.anu.edu.au/research/projects/australian-national-bushfire-health-and-wellbeing-study-bushfirestudy.

## Footnotes

1 The fire season lasted from July 2019 to May 2020, however, for most people the threats to life and property occurred between November 2019 – February 2020. Our survey was conducted January-March 2021.
